# Short-Term Effects of the 2008 Cold Spell on Mortality in Three Subtropical Cities in Guangdong Province, China

**DOI:** 10.1289/ehp.1104541

**Published:** 2012-10-31

**Authors:** Huiyan Xie, Zhibin Yao, Yonghui Zhang, Yanjun Xu, Xiaojun Xu, Tao Liu, Hualiang Lin, Xiangqian Lao, Shannon Rutherford, Cordia Chu, Cunrui Huang, Scott Baum, Wenjun Ma

**Affiliations:** 1 Guangdong Provincial Institute of Public Health, Guangzhou, Guangdong, China; 2 Guangdong Provincial Department of Health, Guangzhou, Guangdong, China; 3 Center for Disease Control and Prevention of Guangdong Province, Guangzhou, Guangdong, China; 4 School of Public Health and Primary Care, Faculty of Medicine, The Chinese University of Hong Kong, Hong Kong, China; 5 Center for Environment and Population Health, School of Environment, Griffith University, Brisbane, Queensland, Australia; 6 School of Public Health and Institute of Health and Biomedical Innovation, Queensland University of Technology, Brisbane, Queensland, Australia

**Keywords:** climate change, cold spell, mortality, subtropical cities, temperature

## Abstract

Background: Few studies have been conducted to investigate the impact of extreme cold events on mortality in subtropical regions.

Objective: In the present study we aimed to investigate the effects of the 2008 cold spell on mortality and the possibility of mortality displacement in three subtropical cities in China.

Methods: Daily mortality, air pollution, and weather data were collected from 2006 to 2009 in Guangzhou, Nanxiong (no air pollutants), and Taishan. We used a polynomial distributed lag model (DLM) to analyze the relationship between the 2008 cold spell and mortality. To observe the mortality displacement of the cold spell, we estimated the cumulative effects at lag0, lag0–6, lag0–13, lag0–20, and lag0–27 separately.

Results: During the 2008 cold spell, the cumulative risk of nonaccidental mortality increased significantly in Guangzhou [relative risk (RR) = 1.60; 95% CI: 1.19, 2.14] and Taishan (RR = 1.60; 95% CI: 1.06, 2.40) when lagged up to 4 weeks after the cold spell ended. Estimated effects at lag0–27 were more pronounced for males than for females, for respiratory mortality than for cardiovascular mortality, and for the elderly (≥ 75 years of age) than for those 0–64 years of age. Most of the cumulative RRs increased with longer lag times in Guangzhou and Taishan. However, in Nanxiong, the trend with cumulative RRs was less consistent, and we observed no statistically significant associations at lag0–27.

Conclusion: We found associations between the 2008 cold spell and increased mortality in the three subtropical cities of China. The lag effect structure of the cold spell varied with location and the type of mortality, and evidence of short-term mortality displacement was inconsistent. These findings suggest that extreme cold is an important public health problem in subtropical regions.

Climate change is likely to cause increased occurrence of extreme weather events, including both heat waves and cold spells (Molloy et al. 2008). Many studies have examined the relationship between extreme temperature events and mortality (Gómez-Acebo et al. 2010; Hajat et al. 2005; Iniguez et al. 2010; Kaiser et al. 2007; Rooney et al. 1998; Sartor et al. 1995), mainly focusing on heat waves to demonstrate the effects of global warming (Gasparrini and Armstrong 2011; Knowlton et al. 2009; Le Tertre et al. 2006; Semenza et al. 1996; Tong et al. 2010), but fewer studies have examined the health effects of extreme cold spells (Kysely et al. 2009; Montero et al. 2010). As noted by Ballester et al. (2003), some studies have reported greater cold-related mortality than heat-related mortality; in addition, heat wave effects appear to last for a few days at most, whereas effects of cold spells may persist for up to 2 months. Most studies on the impact of extreme cold events have been conducted in temperate cities in developed countries (Analitis et al. 2008; Cagle and Hubbard 2005; Healy 2003; O’Neill et al. 2003). Estimated effects of temperature on mortality may be heterogeneous across areas with differing socioeconomic status and education level (Basu and Samet 2002; Bell et al. 2008). However, few studies have been conducted in tropical or subtropical cities in developing countries.

Guangdong, a subtropical province in China, experienced an unusually persistent and widespread severe cold spell in 2008. This event also affected 20 other provinces across southern China. The daily mean temperature during this extreme weather event was much lower than that for the same period in previous years. Although intensive public attention was focused on the adverse impact of this cold spell on ecological, social, and economic systems, health impacts on local residents have not been studied (Liangxun et al. 2009).

Many previous studies on associations of temperature with mortality have considered delayed effects (Bell et al. 2008; Hajat et al. 2005; Hertel et al. 2009; Huynen et al. 2001; Kysely 2004), including lagged effects of temperature on single days, and of moving average temperature on subsequent days. For example, Bell et al. (2008) estimated the association between high temperature and mortality using single day lags of 0, 1, 2, and 3 days and cumulative lags up to 1 week (lags 0–6) using a moving average. This approach could overestimate the effects of current-day exposure by ignoring effects of exposure on previous days (Gasparrini et al. 2010). However, it may also underestimate effects of exposure on mortality if effects persist longer than the observed lag period (Roberts and Martin 2007; Schwartz 2000). Distributed lag models (DLMs), which allow a detailed representation of the time course of the exposure–response relationship while avoiding problems related to colinearity among lagged exposure variables, have been proposed for analyses of delayed effects (Schwartz 2000). Numerous studies have applied DLMs to analyze lagged health effects of temperature, primarily for continuous-temperature time-series analysis (Analitis et al. 2008; Ha et al. 2011; Hajat et al. 2005; Liu et al. 2011).

In the present study we aimed to assess the health impacts of the 2008 cold spell in three subtropical cities of Guangdong by analyzing extended time-series data for daily mortality and modeling lagged effects using distributed lag models. The findings of this study will improve our understanding of relationships between extreme cold events and mortality in subtropical areas and provide evidence to support the need to develop adaptation strategies to mitigate the adverse effects of cold climate extremes in the context of climate change.

## Materials and Methods

**Figure 1:**
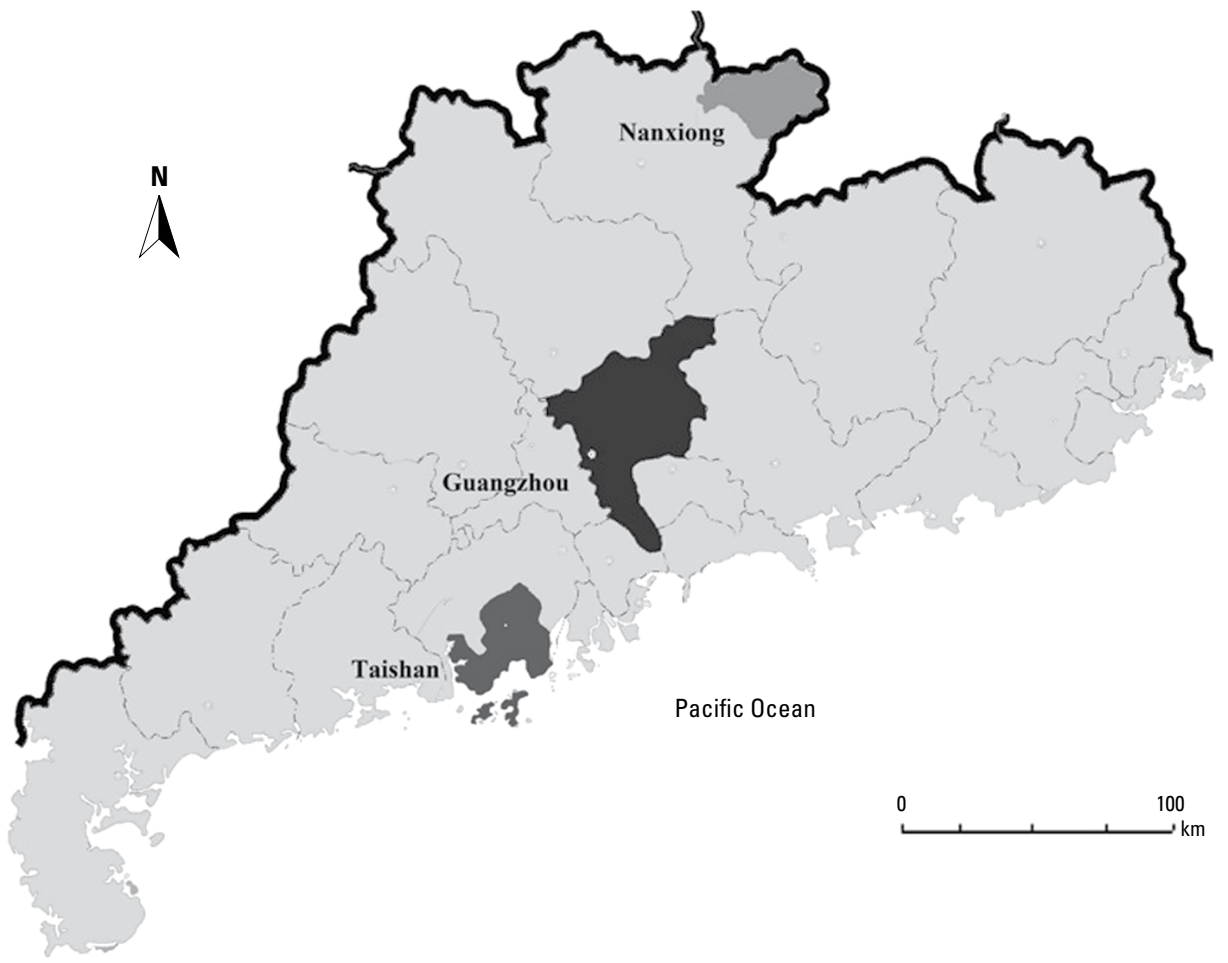
Map of Guangdong Province, China, highlighting the cities of Nanxiong, Guangzhou, and Taishan.

*Study settings.* Guangdong is one of China’s southernmost provinces. It has a typical subtropical climate with an average annual temperature of 22°C. Data were collected for three cities located in different parts of the province (Figure 1): Nanxiong, the northernmost city, with a population of > 400,000 by the end of 2009; Guangzhou, the centrally located capital of Guangdong Province, with a total population of > 7 million; and Taishan, a coastal city in southern Guangdong, with a population > 900,000 by the end of 2009. On the basis of data availability, we used data from two districts of Guangzhou (Yue Xiu and Li Wan, with an estimated population of 1.86 million in 2009) for this study.

*Data sources*. Daily nonaccidental mortality data from 1 January 2006 through 31 December 2009 were obtained from the Guangdong Provincial Center for Disease Control and Prevention. The original data source was death certificates, which included the age and sex of the deceased and the date and cause of death. Nonaccidental causes of deaths were categorized using codes A00–R99 from the *International Classification of Diseases*, *10th Revision* (ICD–10; World Health Organization 2007). ICD-10 codes J00–J99 represent respiratory diseases, and codes I00–I99 represent cardiovascular diseases.

Daily meteorological data were collected from the local meteorological bureaus of each city from 1 January 2006 through 31 December 2009. We used maximum temperature, mean temperature, minimum temperature (T_min_), and relative humidity (RH) for the analysis.

Air pollution data obtained for the same period included daily average concentrations of particulate matter with aerodynamic diameter < 10 μm (PM_10_), nitrogen dioxide (NO_2_), and sulfur dioxide (SO_2_), all given in milligrams per cubic meter. Air pollution data were measured at single environmental monitoring sites located in the centers of Guangzhou (monitored continuously) and Taishan (monitored hourly). Air pollution data were not available for Nanxiong.

*Definition of cold spell*. A variety of approaches have been used to define a cold spell (Kysely et al. 2009; Lin et al. 2011; Montero et al. 2010), but there is no universally accepted definition based on specific temperatures. The Chinese National Bureau of Meteorology defines a cold spell as a period with a temperature decrease of at least 8°C over 48 hr that results in a T_min_ < 4°C (China Meteorological Administration 2008). However, this definition was inappropriate for Guangdong because the province is a subtropical region. Because the T_min_ of the three cities correlated more closely with their respective mortality than did maximum temperature and mean temperature (results not shown), the definition of cold spell that we adopted for this analysis was based on the daily T_min_. Therefore, in this study, we defined a weather fluctuation as a cold spell if the minimum daily temperature fell below the 5th percentile of temperatures recorded at that location from January 2006 through December 2009 for at least 5 consecutive days. This definition was very similar to that used in a previous meteorological study in China (Wu et al. 2008). According to this definition, the 2008 cold spell lasted between 18 and 21 days in the three sampled Guangdong cities. Table 1 shows detailed information on this cold spell in these three cities.

**Table 1:** Threshold temperatures (T_min_) and their durations for the 2008 cold spell in three cities in Guangdong Province, China.

City (location)	Threshold temperature (°C)	Cold spell dates
Guangzhou (23°16´N,113°14´E)	6.3	26 Jan 2008–15 Feb 2008
Nanxiong (25°14´N, 114° 33´E)	2.1	25 Jan 2008–16 Feb 2008
Taishan (22°15´N, 112° 48´E)	7.0	26 Jan 2008–12 Feb 2008

### Statistical Analysis

*Calculation of excess mortality*. To estimate excess mortality attributable to the 2008 cold spell, we calculated 31-day moving averages of daily mortality during the cold spell and during the same days in the 2 years before the cold spell and the year after the spell combined (Rooney et al. 1998). Excess mortality was assessed as the difference between the number of deaths observed on a given day during the 2008 cold spell and the corresponding moving average values for 2006, 2007, and 2009 combined. We calculated an approximate conﬁdence interval (CI) for the excess mortality by treating the total number of deaths during the cold spell as a Poisson distribution and comparing the upper and lower 95% conﬁdence bounds of this value with the expected number of deaths.

*Estimation of city-specific relative risk*. We evaluated the association between the 2008 cold spell and daily mortality using Poisson regression with a distributed lag model. For Poisson regression, the unconstrained distributed lag model may be written as

Log(μ*_t_*) = α + *COV*s + β_0_*Z_t_* + β_1_*Z_t_*_– 1_ + … + β*_j_ Z_t – q_*, [1]

where *COV*s represents all other covariates in the model, and *Z_t_* represents cold-spell exposure delayed over time for *j* = 0 … *q* days. In this study, we defined *Z_t_* as a binary variable that equals 1 for the 2008 cold spell days and 0 for other days.

To gain more precision in the estimate of the distributed lag curve, a polynomial distributed lag constrains the β*_j_* to follow a polynomial pattern in the lag number:


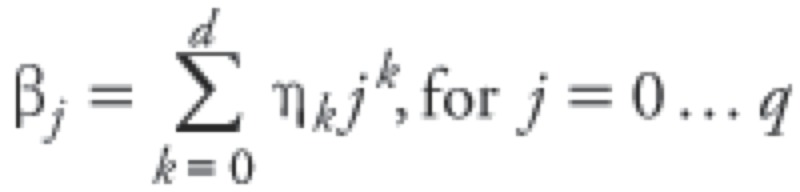
[2]

where *j* is the number of lag days and *d* is the degree of the polynomial. We chose to use a third-degree polynomial in this study to ensure enough degrees of freedom (df) to fit the pattern of response over time. We specified the lagged effect of the cold spell up to 27 days, consistent with previous studies (Armstrong 2006; Guo et al. 2011).

We estimated the cumulative mortality risk associated with the cold spell using the cross-basis functions for the spaces of the cold spell and the lag dimension [based on a bidimensional functional space expressed by the combination of two sets of basis functions, which specify the relationships in the dimensions of the cold spell and lags (Gasparrini 2011)] as a covariate in the Poisson regression model. Cumulative mortality risk and 95% CIs were estimated by comparing mortality during the cold spell with mortality during the non–cold spell periods. To observe mortality displacement, we estimated cumulative effects for lag 0, lag 0–6, lag 0–13, lag 0–20, and lag 0–27 days.

Relative humidity, PM_10_, NO_2_, and SO_2_ were modeled as natural cubic splines with 3 df in models for Guangzhou and Taishan, as described previously (Anderson and Bell 2009; Guo et al. 2011). However, we did not adjust for air pollutants in Nanxiong because air pollutant data were not available. We also modeled a binary variable assigned as 1 on days when any influenza deaths were reported and 0 otherwise (ICD-10 codes J10–J11) to account for influenza viral activity, similar to the approach used by Braga et al. (2000). To control for seasonality and long-term trends, we included a smooth function of time and also included day of the week as a covariate in the models. Therefore, the complete Poisson regression model was

*Y_t_*~Poisson (μ*_t_*) Log(μ*_t_*) = α + β_0_*Z_t_* + β_1_*Z_t_*_– 1_ + … + β*_j_Z_t – q_* + *S*(RH*_t_*, 3) + *S*(PM_10_*_t_*, 3) + *S*(SO_2_*_t_*, 3) + *S*(NO_2_*_t_*, 3) + *S*(Time,8/year) + ηDOW*_t_* + υInfluenza*_t_*      = α + β_0_*Z_t_* + β_1_*Z_t_*_– 1_ +…+ β*_j_Z_t – q_* + *COV*s. [3]

Here, *t* is the date of the observation; *Y_t_* is the observed daily death count on day *t;* α is the intercept; *Z_t_* is the cold spell exposure on the same day (lag0), with *Z_t_*_– 1_ on the previous day (lag1), and so on. *S*() is a natural cubic spline. RH*_t_*, PM_10_*_t_*, SO_2_*_t_*, and NO_2_*_t_* represent the relative humidity and concentrations of PM_10_, NO_2_, and SO_2_, respectively, at time *t* with 3 df; *S*(time,8/year) is the natural cubic spline of time with 8 df per year, which was chosen by minimizing the Akaike information criterion (AIC; Akaike 1974). DOW*_t_* is the day of the week on day *t*, and η is the vector of coefficients. Influenza is a binary variable that is 1 if there are any influenza deaths on day *t* and 0 if there are not.

All statistical tests were two-sided, and values of *p* < 0.05 were considered statistically significant. We used R software (version 2.11.0; R Development Core Team, http://www.R-project.org/) and SAS software (version 9.1; SAS Institute Inc., Cary, NC, USA; Daly 1992) to analyze the data. The dlnm package in R software was used to construct the polynomial distributed lag basis.

## Results

Table 2 summarizes the weather, air pollutant, and mortality statistics during the 2008 cold spell and the corresponding days during 2006, 2007, and 2009 in Guangzhou, Nanxiong, and Taishan. Compared with the same periods in 2006, 2007, and 2009, the mean daily T_min_ during the 2008 cold spell was > 7°C lower, with the lowest mean daily T_min_ reaching 2.26°C in Nanxiong. Mean daily death counts during 2006, 2007, and 2009 of 37, 7, and 23 in Guangzhou, Nanxiong, and Taishan, respectively, increased to 55, 11, and 32 during the 2008 cold spell.

**Table 2:** Comparison of weather, air pollution, and mortality rates between the 2008 cold spell and the corresponding days in 2006, 2007, and 2009 in three cities in Guangdong Province, China.

City	Populationa	Age > 65 (%)b	2008 cold spell [mean (SD)]						Same days during 2006, 2007, and 2009 [mean (SD)]					
			Tmin (°C)	RH (%)	PM10 (μg/m3)	SO2 (μg/m3)	NO2 (μg/m3)	nc	Tmin (°C)	RH (%)	PM10 (μg/m3)	SO2 (μg/m3)	NO2 (μg/m3	nc
Guangzhou	1,869,790	11.12	6.06 (1.40)	67.90 (21.34)	88.10 (27.12)	68.78 (21.98)	87.55 (36.34)	54.48 (7.18)	13.69 (3.47)	69.17 (6.54)	75.73 (11.59)	50.25 (12.27)	68.78 (16.04)	37.09 (4.48)
Nanxiong	474,910	9.32	2.26 (1.33)	71.19 (20.75)	—	—	—	10.74 (2.07)	9.48 (3.71)	70.70 (4.5)	—	—	—	7.00 (1.87)
Taishan	985,863	11.03	6.09 (1.07)	77.78 (15.96)	79.30 (17.91)	71.26 (28.28)	56.89 (32.91)	31.72 (6.34)	13.07 (2.51)	69.85 (5.33)	82.76 (18.94)	55.63 (14.00)	54.25 (12.85)	23.00 (5.03)
aNumber of residents at the end of 2009. bPercentage of the population > 65 years of age. cDaily number of deaths.															

**Figure 2:**
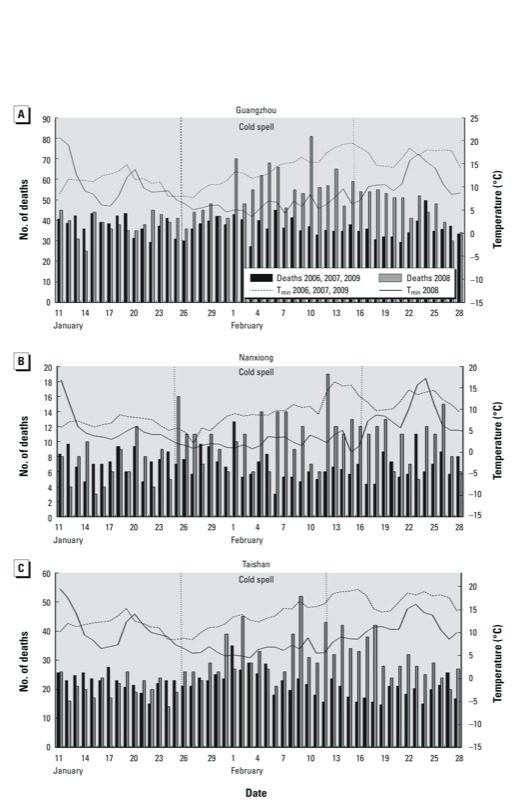
Relationship between T_min_ and daily mortality in Guangzhou (*A*), Nanxiong (*B*), and Taishan (*C*) during the 2008 cold spell relative to corresponding days in 2006, 2007, and 2009.

Figure 2 shows the increase of daily death counts observed in the three cities during the 2008 cold spell relative to the means for corresponding days in 2006, 2007, and 2009. The largest increase in mortality was observed in Nanxiong, with 52% more deaths than the average for the corresponding days in 2006, 2007 and 2009, and the smallest increase in deaths was observed in Taishan, with 35% more deaths than in 2006, 2007, and 2009 (Table 3). The excess mortality rate increased dramatically with age in all three cities, and was highest for residents > 75 years of age in Nanxiong (427.2 excess deaths per 100,000; 95% CI: 336.6, 543.7).

**Table 3:** Estimated increases in mortality during the 2008 cold spell compared with the 31-day moving average for the corresponding days in 2006, 2007, and 2009 in three cities in Guangdong Province, China, by age, sex, and cause of death.

Mortality	Guangzhou		Nanxiong		Taishan	
	Excess mortality ratea (95% CI)	Percentb	Excess mortality rate (95% CI)	Percent	Excess mortality rate (95% CI)	Percent
All	18.8 (16.8–20.7)	42.7	17.9 (12.7–23.0)	52.1	15.1 (11.0–19.2)	35.3
Age group (years)
0–64	1.7 (1.1–2.4)	16.5	–1.4 (–4.7–2.1)	–9.6	–0.5 (–2.7–1.5)	–5.8
65–74	29.9 (20.8–39.1)	23.2	87.6 (45.6–129.7)	69.5	24.1 (1.9–61.2)	24.2
≥ 75	248.1 (219.2–277.0)	59.4	427.2 (336.6–543.7)	96.6	258.7 (207.7–320.7)	57.8
Sex
Male	23.8 (20.7–26.9)	50.3	16.4 (8.2–24.2)	42.2	18.1 (12.1–23.9)	40.1
Female	13.9 (11.3–16.1)	33.7	19.4 (12.4–26.4)	64.3	11.1 (6.4–17.6)	29.9
Cause of death
Respiratory diseases	3.1 (2.3–3.9)	39.5	8.2 (5.3–10.8)	87.6	4.4 (2.8–5.8)	78.8
Cardiovascular diseases	10.64 (9.2–12.1)	66.5	8.7 (5.3–11.8)	66.2	10.4 (7.1–13.5)	39.7
aCalculated as the difference between the number of deaths observed during the 2008 cold spell and expected mortality (the corresponding moving average value for 2006, 2007, and 2009 combined) and expressed as a rate of excess deaths in the local population by the end of 2009 in deaths per 100,000. bCalculated as the percentage increase above expected mortality.								

Table 4 presents the cumulative relative risks (RRs) for lag0–27 days by age group, sex, and cause of death. There was a significant increase in nonaccidental mortality during the 2008 cold spell for all ages combined in Guangzhou and Taishan, both before and after adjustment for air pollution, but the increase in mortality in Nanxiong was not statistically significant for any age group or according to sex or cause of death (*p* > 0.05). In Guangzhou and Taishan, the estimated mortality associated with the 2008 cold spell was higher for males than for females (RR = 1.56; 95% CI: 1.07, 2.28 vs. RR = 1.31; 95% CI: 0.85, 2.02 in Guangzhou; RR = 1.93; 95% CI: 1.14, 3.27 vs. RR = 1.27; 95% CI: 0.71, 2.27 in Taishan), for respiratory mortality than for cardiovascular mortality (RR = 2.15; 95% CI: 1.11, 4.16 vs. RR = 1.40; 95% CI: 0.87, 2.25 in Guangzhou; RR = 3.23; 95% CI: 1.38, 7.58 vs. RR = 1.67; 95% CI: 1.02, 2.73 in Taishan), and for those ≥ 75 years of age than for those 0–64 years of age (RR = 1.48; 95% CI: 1.01, 2.17 vs. RR = 1.42; 95% CI: 0.77, 2.60 in Guangzhou; RR = 2.19; 95% CI: 1.34, 2.60 vs. RR = 1.10; 95% CI: 0.50, 2.44 in Taishan).

**Table 4:** Estimated cumulative effects [RR (95% CI)] of the 2008 cold spell on mortality for lag 0–27 days in three cities in Guangdong, China, by cause of death, sex, and age group.

	Guangzhou		Nanxiong	Taishan	
	Model 1a	Model 2b	Model 1	Model 1	Model 2
Age (years)
All	1.60 (1.19, 2.14)*	1.44 (1.08, 1.94)*	1.55 (0.77, 3.11)	1.72 (1.17, 2.55)*	1.60 (1.06, 2.40)*
0–64	1.47 (0.80, 2.68)	1.42 (0.77, 2.60)	1.97 (0.74, 5.25)	1.13 (0.53, 2.44)	1.10 (0.50, 2.44)
65–74	1.85 (0.97, 3.51)	1.69 (0.88, 3.23)	1.82 (0.65, 5.09)	0.90 (0.40, 2.01)	0.99 (0.50, 1.95)
≥ 75	1.53 (1.05, 2.24)*	1.48 (1.01, 2.17)*	1.09 (0.41, 2.92)	2.42 (1.50, 3.90)*	2.19 (1.34, 3.60)*
Sex
Male	1.70 (1.17, 2.48)*	1.56 (1.07, 2.28)*	1.46 (0.62, 3.43)	1.87 (1.13, 3.09)*	1.93 (1.14, 3.27)*
Female	1.47 (0.96, 2.26)	1.31 (0.85, 2.02)	1.71 (0.71, 4.11)	1.55 (0.89, 2.70)	1.27 (0.71, 2.27)
Cause of death
Respiratory diseases	2.33 (1.22, 4.46)*	2.15 (1.11, 4.16)*	1.53 (0.63, 3.68)	3.38 (1.54, 7.41)*	3.23 (1.38, 7.58)*
Cardiovascular diseases	1.59 (0.99, 2.55)	1.40 (0.87, 2.25)	0.72 (0.28, 1.85)	1.73 (1.06, 2.83)*	1.67 (1.02, 2.73)*
aAdjusted for RH, seasonality and long-term trends, day of the week, and influenza deaths. bAdjusted for RH, seasonality and long-term trends, day of the week, influenza deaths, and air pollution. *p < 0.05.							

**Figure 3:**
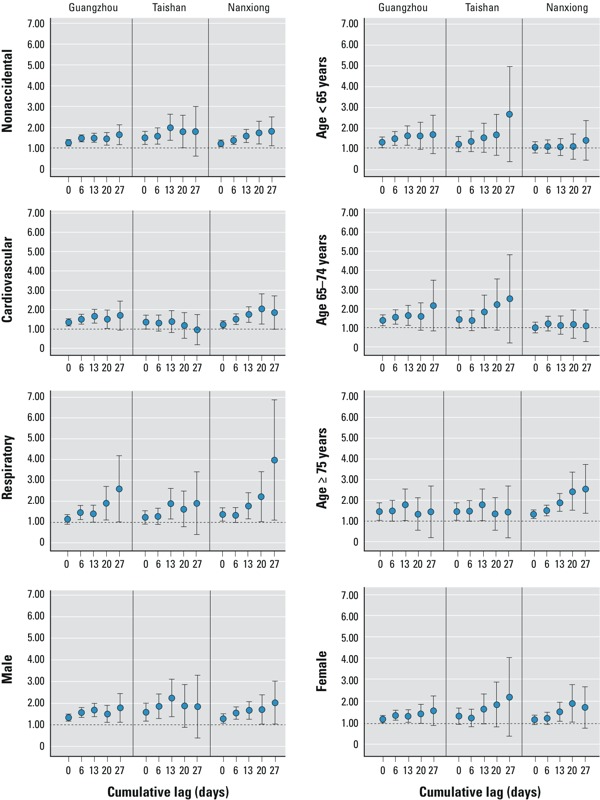
City-specific cumulative RRs (95% CIs) of mortality in three cities in Guangdong, China, during the 2008 cold spell, by cause of death, sex, and age group using dlnm for different lag days, with adjustment for RH, seasonality and long-term trends, day of the week, and influenza deaths.

To evaluate the lag structure of effects of the cold spell on mortality, including potential effects of mortality displacement, we estimated cumulative effects by age group, sex, and cause of death for different lags using the distributed lag model (Figure 3). The cumulative RRs based on these analyses can be interpreted as the net effects of the cold spell after accounting for mortality displacement, which is characterized by an increasing trend of cumulative RRs for exposures at lower lags (resulting in part from deaths that occurred earlier in time as a consequence of exposure) followed by a decline in cumulative RRs at higher lags (because of the relative deficit in deaths that have been displaced forward in time) (Hajat et al. 2005; Roberts and Switzer 2004). In general, RRs were lowest at lag0; in Guangzhou and Taishan, cumulative RRs increased with longer cumulative lags, with the highest RR at lag27. However, in Nanxiong, the highest cumulative RRs (except for those affecting residents < 75 years of age or females) were observed at lag0–13, after which they decreased slowly, suggesting a deficit offset for only part of the overall excess after 2 weeks of exposure.

## Discussion

In view of the global change in climate predicted for future decades, the frequency, intensity, and duration of extreme climate events are expected to change (Albritton et al. 2001). Understanding the relationship between extreme climate events, such as heat waves and cold spells, and their potential health impacts is the first step in managing and reducing the adverse impact of such events. To our knowledge, the present study is unique in estimating the short-term effects of a cold spell on mortality in multiple subtropical cities in China using distributed lag models.

Our estimates of increased mortality during the 2008 cold spell are much higher than those from studies in the Netherlands (Huynen et al. 2001), Russia (Revich and Shaposhnikov 2008), or the Czech Republic (Kysely et al. 2009). There are several possible reasons for this. First, techniques used to measure increased mortality varied across studies. For example, different reference baselines can lead to different estimated values for increased mortality. For example, Kysely et al. (2009) calculated the expected (baseline) number of deaths using the mean annual cycle smoothed by 15-day running means adjusting for the observed year-to-year changes in mortality. Second, populations who live in cold climates may be more accustomed to and prepared for extreme cold weather than subtropical residents. The 2008 cold spell that occurred in Guangdong was popularly considered to be the most extreme cold spell in five decades, and hence populations of these three subtropical cities may be more sensitive to extreme cold weather (Analitis et al. 2008). Third, in subtropical areas such as Guangdong Province, few buildings have heating systems equipped to provide enough heat in extremely cold weather; this may contribute to a greater risk for vulnerable populations, such as the elderly. Finally, the health care system, especially the emergency service, could not meet the sudden increase in the need for care during the extreme cold event in southern China, when the number of ambulance calls rose to such a high number that nearly one-fourth of the calls received no response (Xiong et al. 2010).

Our findings suggest that the elderly suffered the most during the 2008 cold spell, with those > 75 years of age being the most susceptible, consistent with some previous studies (Analitis et al. 2008; Huynen et al. 2001; Iniguez et al. 2010; Revich and Shaposhnikov 2008). However, our findings differed from a study conducted in the Czech Republic, in which cold spells had the greatest effect on middle-aged men who died from cardiovascular disease (Kysely et al. 2009). The authors attributed this finding to occupational exposure to the cold in men 25–59 years of age, whereas the elderly tended to stay indoors during the cold spell and thus avoided direct exposure to ambient temperatures.

We also found that the effects of the cold spell appeared to be more pronounced for respiratory disease patients than that for cardiovascular patients, consistent with previous findings from Russia (Revich and Shaposhnikov 2008) and Europe (Analitis et al. 2008). Some researchers have attributed the increased deaths from respiratory diseases to increased infection from indoor crowding, adverse effects of cold weather on the immune system, and the fact that low temperatures may facilitate the survival of bacteria in droplets (The Eurowinter Group 1997; Handley and Webster 1995).

In the present study, the effects of the 2008 cold spell on nonaccidental mortality appeared to be greater within 2 weeks of exposure in Nanxiong compared with the other two cities. One possible explanation for this is that Nanxiong is the northernmost city of the three cities and had the lowest average T_min_ during the 2008 cold spell. Another possible reason is a lower adaptive capacity to extreme weather events in Nanxiong, as reflected by the lower socioeconomic status of the city, for which gross national product per capita was much lower than in the other two cities. To explore this further, it would be necessary to study the social determinants of adaptive capacity for extreme weather events.

We also found an apparent rise in respiratory disease mortality that lasted up to 4 weeks after the cold spell. This was consistent with two studies in which the apparent effects of low temperatures continued for a longer period of time than did estimated effects of heat waves (Healy 2003; Iniguez et al. 2010). In Nanxiong and Guangzhou, estimated effects decreased after 2 weeks of the cold spell for persons > 75 years of age, suggesting some compensatory risk reduction consistent with a harvesting phenomenon (Hajat et al. 2005). However, this phenomenon was not observed in Taishan. This discrepancy needs to be explored further.

We controlled for air pollution effects in Guangzhou and Taishan (pollution data were not available for Nanxiong), and the estimated effects of the cold spell showed a slight reduction after adjustment. This might be explained by the relative impacts of air pollution on mortality during the cold spell, because air pollutant concentrations are likely to increase during such episodes (O’Neill et al. 2005; Schwartz 2000; Tong et al. 2010). Previous studies have suggested that the effects of air pollution on mortality are much lower than the effects of temperature (Ren et al. 2011; Spickett et al. 2011). Thus, the relationship we observed between mortality and the cold spell was not likely to have been substantially confounded by the effects of air pollution. However, we did not consider the possible effects of indoor air pollution from smoking, cooking, and home-heating fuels on mortality.

In the present study, we used a binary indicator to describe both cold-spell and non–cold-spell days to attain unique DLM coefficients representing overall mortality effects of the cold spell period. This approach is different from modeling mortality as a continuous function of temperature, as has been done in previous studies (Hertel et al. 2009; Kaiser et al. 2007). Although we believe these approaches are comparable from a purely conceptual viewpoint, the validity of our approach should be evaluated in future studies.

Our findings suggest that further research is needed. First, studies should be conducted based on longer time-series data with multiple cold spells to estimate the impact of cold spells according to their duration or intensity. Second, the estimated effects of weather on mortality may have been influenced by the age structure, sociodemographic characteristics, and environmental conditions of each population. Further research on factors that determine vulnerability to cold would help inform the development and implementation of cold-weather emergency plans. Information on the effects of indoor environments, energy usage, and human thermal comfort thresholds on vulnerability would also help determine appropriate strategies for adapting to a changing climate.

## Conclusion

The 2008 cold spell was associated with an increase in daily mortality in three subtropical cities of Guangdong Province, China. As a subtropical region, Guangdong is relatively ill equipped to adapt to extreme cold events. For example, most temperature control systems in buildings in Guangdong Province were designed for cooling, not heating. Climate models indicate that seasonal weather patterns and conditions will continue to vary from current climate conditions as average global temperatures increase (Albritton et al. 2001), and climate change is expected to contribute to an increase in the intensity of extreme cold events as well as heat waves (Lionello et al. 2008). It is both necessary and timely for governments and relevant sectors to develop adaptive plans for such extreme events. Similar to the heat-watch warning system adopted in the United States (Kalkstein 2000), subtropical cities need to develop cold weather emergency plans to improve the delivery of health emergency services, and also to issue timely weather alerts when extreme events are expected. On the basis of findings in this study, decision makers from subtropical regions not only should pay attention to heat waves but also must consider adaptive measures to protect vulnerable populations from extreme cold events.
